# Burden and trends of cardiovascular diseases attributable to high LDL-C in China, 1990–2021: an age-period-cohort analysis with projectioans to 2050

**DOI:** 10.3389/fcvm.2025.1698172

**Published:** 2025-11-26

**Authors:** Jingxiao Li, Yuchen Gao, Mengyue Ren, Zihui Yu, Xiaohui Ren, Siwei Chao, Yudong Wu, Mingyue Ma, Yu Wang

**Affiliations:** 1 Department of Epidemiology and Health Statistics, Shenyang Medical College, Shenyang, Liaoning, China; 2 Shenyang Medical College, Shenyang Medical College, Shenyang, Liaoning, China; 3 School of Public Heath, Shenyang Medical College, Shenyang, Liaoning, China

**Keywords:** low-density lipoprotein cholesterol, cardiovascular disease, age-period-cohort model, disease prediction, China

## Abstract

**Objective:**

To analyze the changing trend of the burden of cardiovascular diseases (CVD) caused by high low-density lipoprotein cholesterol (LDL-C) among Chinese residents from 1990 to 2021, as well as the variations in CVD burden by age, period, and cohort, and to predict the trend of CVD burden in 2050, providing a reference for the formulation of CVD prevention strategies in China.

**Methods:**

Data on CVD caused by high LDL-C in China from 1990 to 2021 were collected from the Global Burden of Disease (GBD) database. Age-standardized mortality rate (ASMR) and age-standardized disability-adjusted life years rate (ASDR) were obtained to assess the burden of CVD. The Joinpoint model was used to calculate the average annual percentage change (AAPC) of CVD burden and analyze the changing trends of disease burden by gender. The Age-Period-Cohort (APC) model was employed to explore the relationships among age, period, and cohort factors in CVD burden. The Auto Regressive Integrated Moving Average (ARIMA) model was applied to predict the trend of CVD burden in 2050.

**Results:**

In 2021, the total number of CVD deaths caused by high LDL-C in China reached 832,884.39(424,042.39–1,289,266.00), a significant increase of 194.37% compared to 1990. The disability-adjusted life years (DALYs) were 18,407,759 (95% UI: 10,268,061–27,106,771), a substantial increase of 132.86% compared to 1990. ASMR slightly increased from 43.10 (95% UI: 21.29–67.60) per 100,000 in 1990 to 44.97 (95% UI: 23.22–69.89) per 100,000 in 2021, with an AAPC of −0.47 (95% CI −0.83 to −0.10). ASDR decreased from 385.65 (95% UI: 129.83–647.51) per 100,000 in 1990 to 335.59 (95% CI: 112.75–566.25) per 100,000 in 2021, with an AAPC of −0.49 (95% CI −0.83 to −0.10). Stratified by gender, ASMR significantly increased by 9.75% in men (from 48.82 per 100,000 to 58.57 per 100,000), while it decreased by −3.96% in women (from 38.91 per 100,000 to 34.95 per 100,000). The burden of disease increased sharply with age from 1990 to 2021. The period effect showed that mortality and DALYs increased from 1990 to 2009 and then decreased after 2010. The cohort effect indicated that women born before 1940 had a higher burden, while the risk for men continued to rise after 1940. The ARIMA model's prediction indicates that by 2050, the age-standardized mortality rate (ASMR) of CVD caused by high LDL-C in China is expected to increase by 55.14%, and the age-standardized death rate (ASDR) will rise to 1873.18 per 100,000.

**Conclusion:**

The burden of CVD caused by high LDL-C in China increased overall from 1990 to 2021. The stratified results by gender indicated that the ASMR and ASDR of men were higher than those of women. Stratification by age showed that the burden was the greatest among people over 70 years old. This was mainly due to population growth, aging, and risk factors. The prediction shows that the disease burden will continue to rise until 2050, and men will remain a high-risk group. Given the continuous trend of population aging, it is necessary to consider various lipid-lowering strategies and the effective allocation of medical resources in the future to reduce the disease burden related to CVD caused by high LDL-C.

## 1 Introduction

Cardiovascular disease (CVD) has become one of the leading causes of the global disease burden. Over the past few decades, both the incidence and mortality rates of cardiovascular disease have shown a significant upward trend. According to GBD, CVD causes more than 19.41 million deaths each year, accounting for 29 percent of global mortality (1). Research results show that the mortality rate of cardiovascular diseases in China remains the highest. The mortality rates of cardiovascular diseases in rural and urban areas are 309.33 per 100,000 and 265.11 per 100,000 respectively, accounting for 45.5% and 43.16% of the total mortality rate respectively. This has brought a huge disease and economic burden to society (2).

The elevation of LDL-C level is a major inducer of atherosclerosis. High LDL-C will promote the deposition of lipids in the arterial wall, drive the formation of plaques and vascular inflammation, and then cause coronary artery stenosis. Timely intervention of LDL-C is an important means to effectively alleviate CVD (3, 4). Over the past few decades, with the rapid global economic development, the rapid advancement of urbanization, changes in people's dietary habits, and the increasing prevalence of sedentary lifestyles, the prevalence of high LDL-C is on the rise (5, 6). A CVD study in China, based on an analysis of approximately 170,000 survey participants, found that the prevalence of high LDL-C was 8.1%; and among individuals at high risk of atherosclerotic cardiovascular diseases, only 5.5% received treatment (7). The study also found that from 2002 to 2015, the prevalence of high LDL-C in China increased from 1.3% to 7.2%, indicating a significant rise in dyslipidaemia (8). A longitudinal study conducted in Iran demonstrated that individuals with LDL-C levels ≥2.59 mmol/L exhibited a significantly elevated risk of developing CVD. This observation suggests that High LDL-C may serve as an independent contributing factor to increased CVD incidence (9). A survey study in North African and Middle Eastern countries found that high LDL-C is among the primary risk factors for cardiovascular disease (10).

Currently, in the field of CVD research, previous studies have analyzed the global burden of CVD and its associated risk factors. However, existing research has examined various attributable risk factors in a generalized manner. Nevertheless, discussions on key risk factors remain insufficient. As one of the most critical target risk factors for intervention in the progression of cardiovascular disease, elevated LDL-C levels warrant further investigation into their long-term impact on disease burden and the corresponding predictive mechanisms.

In this study, we relied on the GBD 2021 database, which covers 204 countries and regions worldwide, includes 371 types of diseases and injuries, and 88 types of risk factor information, providing comprehensive and systematic global health data support for research. During the study, we focused on the role of high LDL-C in the burden of CVD, using the age-period-cohort (APC) model to systematically analyse the temporal evolution of the burden of CVD caused by high LDL-C. Additionally, we employed the ARIMA model to predict the trends in the burden of CVD from 2022 to 2050. We hope that this study will provide data references to lay a solid theoretical foundation for the scientific formulation of CVD prevention strategies and the effective alleviation of health burdens among high-risk populations.

## 2 Methods

### 2.1 Data sources

The available data for the association between high LDL-C and CVD in this study were retrieved from the Global Burden of Disease (GBD) database in 2021 (https://vizhub.healthdata.org/gbdresults/). Specifically, the following strategy was employed to extract relevant data from the Global Burden of Disease (GBD) database: using “China” as the geographic scope, “cardiovascular diseases” as the etiology, and “high low-density lipoprotein cholesterol” as the risk factor. The GBD data takes into account the impact on the survival status of people in different regions, of different ages and of different genders. It provides an integrated dataset, improves the method's performance, uses a single aggregated measure of death and disability, namely DALYs, to quantify and compare the health status of the population, and is currently the most widely adopted and representative method for disease burden assessment and measurement internationally.

### 2.2 Observation indicators

We have compiled data on CVD caused by high LDL-C, including mortality rates, disability-adjusted life years (DALYs), age-standardised mortality rates (ASMR), age-standardised DALYs rates (ASDR), and corresponding 95% uncertainty intervals (UI). Download CVD mortality and DALY data attributed to high LDL-C for different genders from 1990 to 2021, with age groups ranging from 25 to 29, 30–24……90–94, and ≥95 years, with each age group spanning 5 years, totaling 15 age groups. The GBD study adheres to the guiding principles of the “Accurate and Transparent Reporting of Health Estimates” statement (as outlined in the GATHER statement) and provides de-identified and aggregated data without requiring ethical approval (11).

### 2.3 Statistical methods

1. After preliminary data processing using Excel 2019, Joinpoint regression model analysis was conducted using Joinpoint 4.9.0.0 to analyze the time trend of CVD burden caused by high LDL-C from 1990 to 2021. The Joinpoint regression model is a statistical technique designed to identify points of trend transition in time-series data. Its methodology is grounded in piecewise linear regression, which operates under the assumption that data trends may shift at specific time points. The primary objective of this analysis is to precisely locate these moments of slope change, referred to as “joinpoints” (12). To establish “joinpoints”, data preparation and configuration are required. It begins with data preprocessing, which involves specifying dependent and independent variables, as well as defining grouping strategies. Subsequently, parameter boundaries are established, and a comprehensive grid search is employed to evaluate all potential joinpoint locations. Furthermore, The significance of identified joinpoints is assessed using metrics such as the Bayesian Information Criterion (BIC), ensuring that detected trend transitions possess statistical validity (13).

The annual percentage change (APC) and average annual percentage change (AAPC) and their 95% confidence intervals (CI) were calculated for each time period. The APC was used to quantify the short-term trends of ASMR and ASDR, thereby clarifying the characteristics of the disease burden changes within specific time periods; while the AAPC served as a measure of the long-term trend, used to assess the overall changes in disease burden from 1990 to 2021. If both APC/AAPC and their 95% CI were >0, it indicated a significant upward trend during that period. Conversely, if both were <0, it indicated a significant downward trend. A *P* value <0.05 was considered statistically significant.

1. The age-period-cohort (APC) model was used to explain the effects of age, period, and cohort on the CVD burden caused by high LDL-C. The age effect refers to the differences in outcome variables among different age groups, the period effect indicates the changes in the CVD burden caused by high LDL-C across all age groups over time, and the cohort effect reflects the differences between different groups with the same birth year (14). The data requirements for the APC model are that the age and period intervals must be equal, with data intervals of consecutive 5 years, dividing the age groups from 25 to 95 into 15 groups: 25–29, 30–34……85–94, ≥95 years old, and 7 period groups: 1990–1994, 1995–1999……2020–2021. The birth cohort = period–age, totaling 21 birth cohorts. A critical challenge in APC models is the perfect collinearity among age, period, and cohort variables. This collinearity results in an identification problem, preventing the estimation of unique age, period, and cohort effects using ordinary least squares or maximum likelihood methods. To resolve this issue, we use the Intrinsic Estimator (IE) method to estimate the effect coefficients for age, period, and cohort.
2. The ARIMA model is a widely used time series forecasting model that integrates autoregressive (AR) and moving average (MA) components, making it suitable for time series variables exhibiting autocorrelation. Its advantages lie in: AR capturing historical burden correlations, integration (I) handling non-stationary trends, and MA counteracting short-term fluctuations, making it well-suited for long-term forecasting; requiring only aggregated data for modeling, unaffected by data gaps or inconsistent diagnostic criteria; With fewer parameters and faster computation, it supports multi-scenario simulations and delivers intuitive results, directly informing policy decisions. Modeling was conducted using SPSS 27. First, enhanced Dickey-Fuller (ADF) tests assessed stationarity; when non-stationary, the difference order was applied to determine the integration order(d). Subsequently, the “Time Series Forecasting - ARIMA” module iterated through combinations of p (0–2) and q (0–2), selecting the optimal model based on the Akaike Information Criterion (AIC). Finally, ACF, PACF, and Ljung-Box tests verified residuals as white noise for predicting age-standardized death rates (ASDRs) over the next 15 years.

## 3 Result

### 3.1 Burden of CVD diseases caused by high LDL-C in different sexes in China from 1990 to 2021

**Figure 1:**
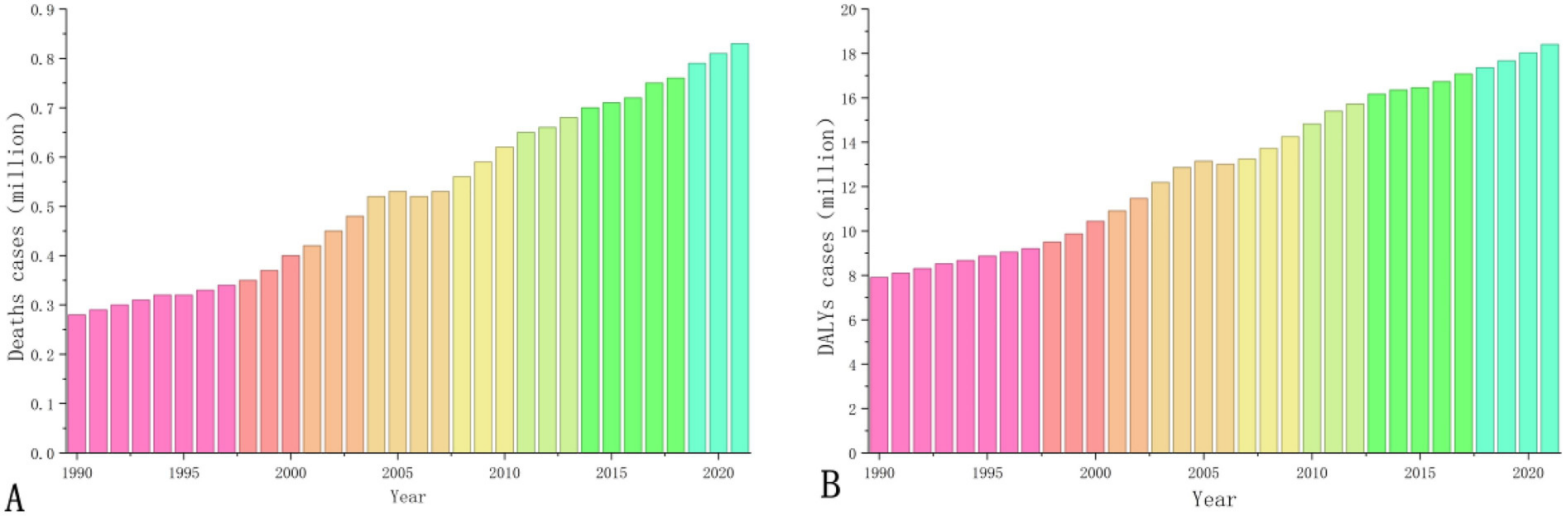
Burden of CVD disease caused by high LDL-C in China, 1990–2021: **(A)** deaths cases; **(B)** DALYs cases.

In 2021, the total number of CVD deaths in China attributable to high LDL-C reached 832,884 (95% UI: 424,042–1,289,266), representing a substantial increase of 194.37% compared to 1990 (Figure 1A). Sex-stratified analysis revealed that female deaths increased by 167.83%, rising from 134,685 (95% UI: 70,198–208,308) to 360,723 (95% UI: 186,199–584,411), while the increase among males was significantly higher at 218.49%, surging from 148,250 (95% UI: 76,802–219,067) to 472,162 (95% UI: 232,650–732,843). The age-standardized mortaliry rates (ASMRs) is generally on the rise (increasing by 1.87%), but there are significant divergences in trends between sexes: the male ASMRs rose markedly by 9.75% (from 48.82/100,000 to 58.57/100,000), whereas the female ASMRs decreased by 3.96% (from 38.91/100,000 to 34.95/100,000) (Table 1).

**Table 1:** Number of deaths and age-standardised mortality rates (ASMRs) from CVD caused by high LDL-C in China in 1990 and 2021.

Gender	Deaths			Age-standardized mortality		
	Number 1990 *n* (95% UI)	Number 2021 *n* (95% UI)	Percentage change, 1990–2021%	Rate in 1990 (per 100,000) %(95% UI)	Rate in 2021 (per 100,000) %(95% UI)	Percent change 1990%–2021%
Both	282935.07 (148118.13,420823.27)	832884.39 (424042.39,1289266.00)	194.37	43.10 (21.29,67.60)	44.97 (23.22,69.89)	1.87
Male	148250.19 (76801.90,219067.49)	472161.65 (232649.94,732842.74)	218.49	48.82 (23.61,75.49)	58.57 (28.56,90.82)	9.75
Female	134684.88 (70198.47,208307.91)	360722.74 (186198.69,584410.71)	167.83	38.91 (19.97,61.86)	34.95 (18.00,56.69)	−3.96

In 2021, the total number of DALYs reached 18,407,759 (95% UI: 10,268,061–27,106,771), representing a remarkable increase of 1,292.82% compared to 1990 (Figure 1B). Sex-stratified analysis indicated that the increase in DALYs was significantly greater among males (1,533.25%) than females (1,040.81%). The ASDR overall decreased by 33.17 per 100,000 (from 953.85 to 920.69 per 100,000). However, distinct sex-based disparities were observed: the age-standardized DALYs rates (ASDRs) among males increased by 120.24 per 100,000 (from 1,072.92 to 1,193.16 per 100,000), while among females, it decreased substantially by 167.20 per 100,000 (from 846.75 to 679.55 per 100,000) (Table 2).

**Table 2:** Number of DALYs and age-standardised disability-adjusted life years rates (ASDRs) for CVD caused by high LDL-C in China in 1990 and 2021.

Gender	DALYs			Age-standardized DALYs		
	Number 1990 *n* (95% UI)	Number 2021 *n* (95% UI)	Percentage change, 1990–2021%	Rate in 1990 (per 100,000) %(95% UI)	Rate in 2021 (per 100,000) %(95% UI)	Percent change 1990%–2021%
Both	7905011.57 (4442434.31,11142363.83)	18407758.53 (10268060.98,27106770.94)	132.86	953.85 (511.60,1397.48)	920.69 (511.71,1359.77)	−33.17
Male	4375007.60 (2402731.02,6203410.37)	11170922.17 (6060075.25,16715660.77)	155.33	1072.92 (561.00,1571.67)	1193.16 (635.37,1787.06)	120.24
Female	3530003.97 (1950253.56,5252967.15)	7236836.36 (3907031.17,11207330.64)	105.01	846.75 (450.67,1277.29)	679.55 (369.17,1050.90)	−167.20

### 3.2 Temporal trends in the CVD burden attributable to high LDL-C by sex in China

**Figure 2:**
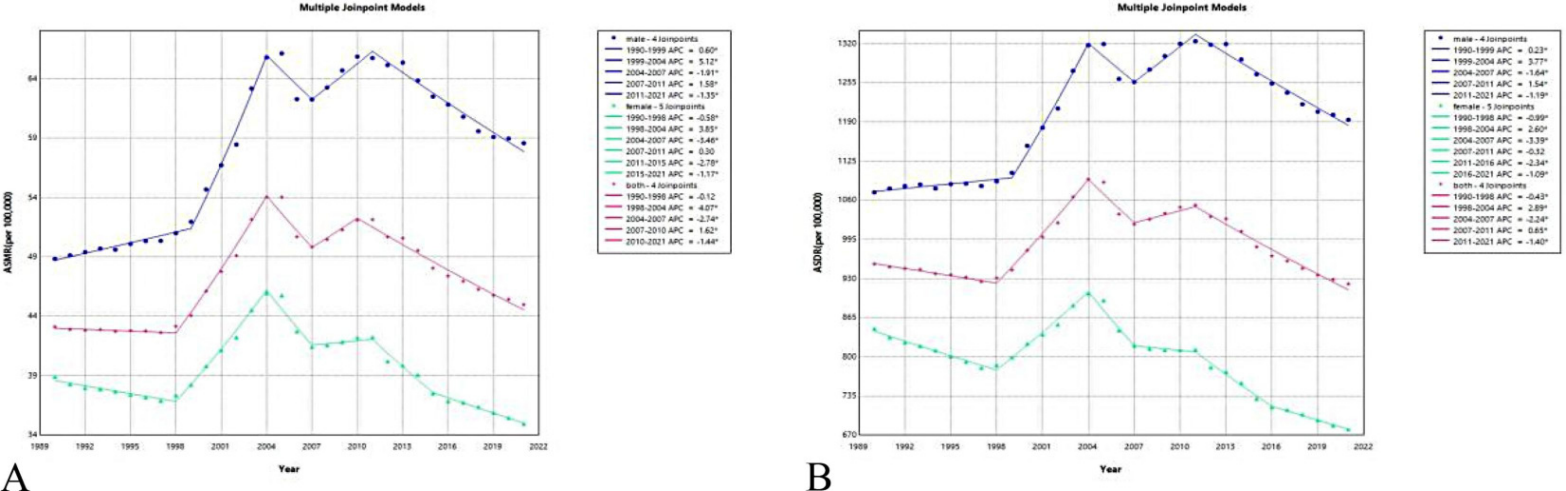
**(A)** ASMRs and **(B)** ASDRs of CVD caused by high LDL-C in China from 1990 to 2021.

Throughout the period from 1990 to 2021, the ASMRs and ASDRs for males consistently remained higher than both the overall population and females. The ASMRs for CVD attributable to high LDL-C exhibited an upward trend among males and the overall population during 1990–2021, while a downward trend was observed among females.The average annual percentage change (AAPC) in ASMRs was 0.56 (95% CI: 0.26–0.86, *p* < 0.001) for males and 0.11 (95% CI: 0.06–0.16, *p* < 0.001) for the overall population. In contrast, the AAPC for females was −0.31 (95% CI: −0.36 to −0.26, *p* < 0.001). Joinpoint regression analysis revealed significant changes in male ASMR in 1999, 2004, 2007, and 2011, while significant changes in female ASMR occurred in 1998, 2004, 2007, 2011, and 2015. Significant change points for the overall ASMR were detected in 1998, 2004, 2007, and 2010 (Figure 2).

Regarding ASDRs, from 1990 to 2021, an upward trend was observed among males, with an AAPC of 0.31 (95% CI: 0.11–0.52, *p* = 0.002). In contrast, downward trends were seen for both females and the overall population, with AAPCs of −0.69 (95% CI: −0.73 to −0.65, *p* < 0.001) and −0.15 (95% CI: −0.19 to −0.12, *p* < 0.001), respectively. Joinpoint regression analysis identified significant change points in ASDRs for males in 1999, 2004, 2007, and 2011. For females, significant change points in ASDRs occurred in 1998, 2004, 2007, 2011, and 2016. Significant change points in the overall ASDRs were detected in 1998, 2004, 2007, and 2011 (Table 3).

**Table 3:** AAPC of ASMR and ASDR caused by high LDL-C in different genders in China.

Gender	ASMR (95% *CI*)	*p*	ASDR (95% *CI*)	*p*
Male	0.56 (0.50,0.61)	<0.001	0.31 (0.27,0.35)	<0.001
Female	−0.31(−0.36,−0.26)	<0.001	−0.69(−0.73,−0.65)	<0.001
Both	0.11 (0.06,0.16)	<0.001	−0.15(−0.19,−0.12)	<0.001

### 3.3 Trends in the burden of CVD attributable to high LDL-C across different age groups in China from 1990 to 2021

**Figure 3:**
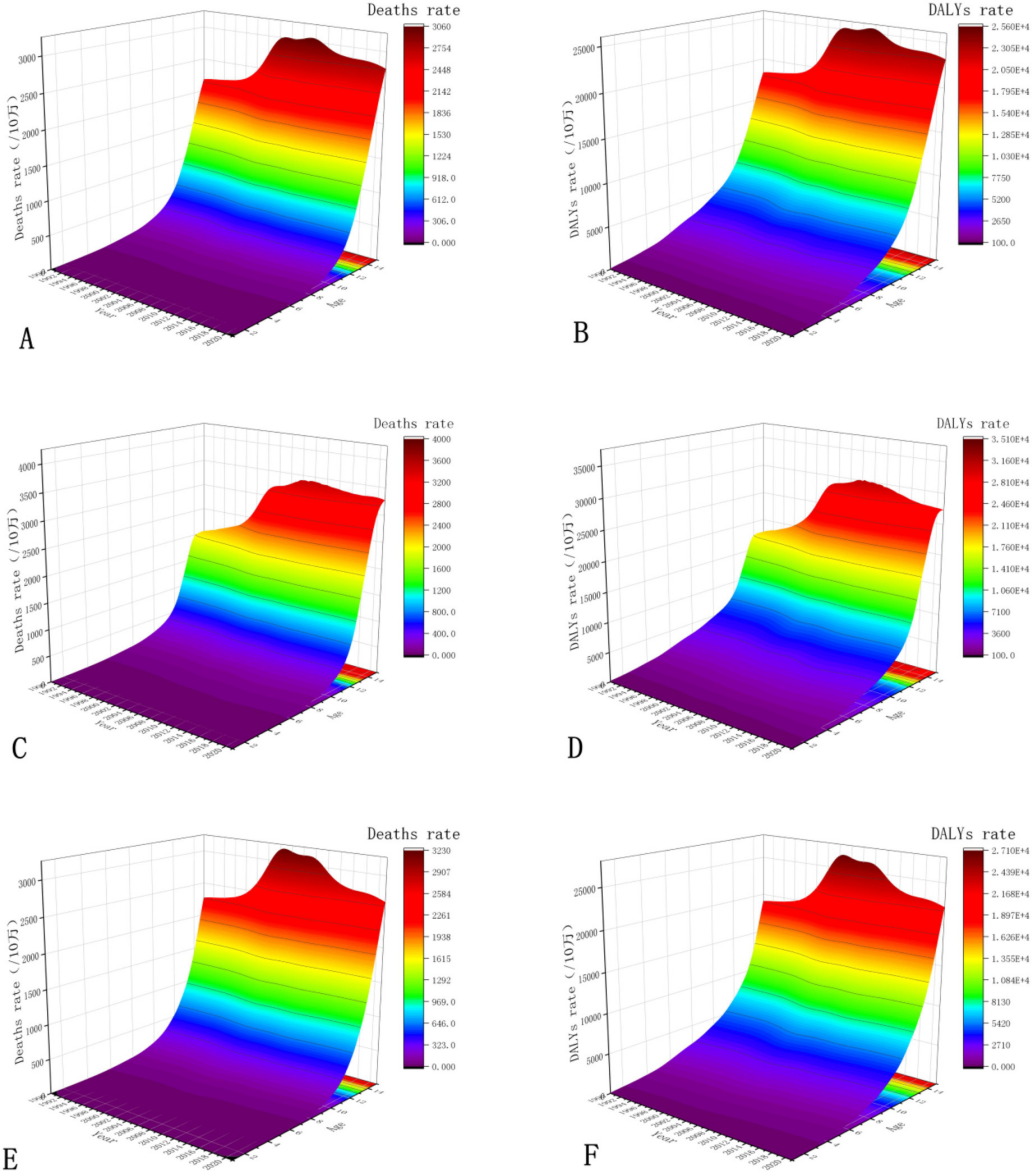
Changes in the disease burden of CVD caused by high LDL-C in different age groups in China from 1990 to 2021. **(A,B)** show the changes in overall standardised mortality rates and standardised DALY rates for different age groups; **(C,D)** show the changes in standardised mortality rates and standardised DALY rates for males in different age groups; **(E,F)** show the changes in standardised mortality rates and standardised DALY rates for females in different age groups.

From 1990 to 2021, the CVD burden attributable to high LDL-C showed an overall increasing trend across different age groups. Mortality rates increased with advancing age, particularly among individuals aged 80 and above. Mortality rates across all age groups showed an overall trend of first increasing and then decreasing over the years, peaking around 2004 and 2011. For instance, among those aged 60–64, the mortality rate increased from 136.50 per 100,000 in 1990 to 117.19 per 100,000 in 2021, with a peak of 154.52 per 100,000 in 2004. In contrast, mortality among those aged 75 and above demonstrated a consistent year-on-year increase (Figure 3A). For example, the mortality rate in the 90–94 age group rose from 1,613.28 per 100,000 in 1990 to 2,100.91 per 100,000 in 2021. A similar upward trend was observed in DALY rates (Figure 3B).

Within the same age group, sex-based disparities were consistently observed across all metrics, with males exhibiting higher values than females. Male mortality rates increased markedly with age, and the rate of increase accelerated in older groups. For instance, mortality was very low among those aged 25–29 (e.g., 3.40 per 100,000 in 2021) but extremely high in those ≥95 years (reaching 3,248.40 per 100,000 in 2021) (Figure 3C). Similarly, DALY rates among males showed a continuous upward trend with age and were substantially higher than mortality rates across all groups (Figure 3D). Female mortality also increased with age but remained consistently lower than that of males within the same age group. For example, in 2021, the mortality rate among females aged ≥95 was approximately 2,628.84 per 100,000, which was lower than that of their male counterparts (Figure 3E). Likewise, DALY rates among females exhibited an upward trend with advancing age (Figure 3F).

### 3.4 1990–2021 age-period-cohort (APC) model analysis of CVD mortality attributable to high LDL-C in China

#### 3.4.1 Age effect

**Figure 4:**
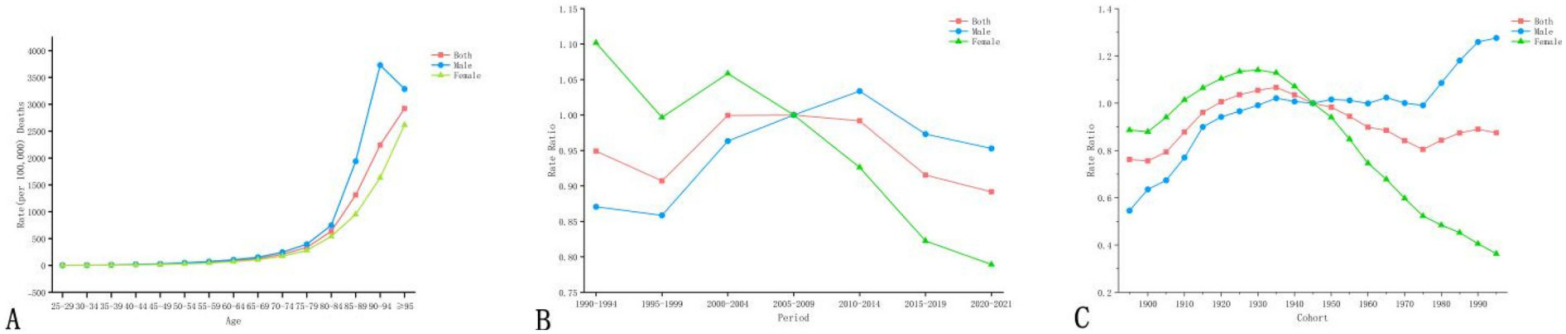
**(A)** Age, **(B)** period, and **(C)** cohort models of CVD mortality attributable to high LDL-C in China, 1990–2021: overall, male, and female.

With increasing age, CVD mortality attributable to high LDL-C in both sexes generally showed an increasing trend, except for males aged 90 years and above, whose mortality rate showed a decreasing trend, as shown in Figure 4A.

#### 3.4.2 Period effects

The overall CVD mortality risk in China showed a pattern of first increasing and then decreasing. Males exhibited a rising trend, whereas females experienced a pronounced decline. Sex-specific disparities demonstrated period-dependent characteristics: during the period from 1990 to 2004, females had relatively higher CVD mortality risk, while after 2009, males showed higher risk (Figure 4B).

#### 3.4.3 Cohort effects

The overall CVD mortality risk among different gender groups in China shows a pattern of first increasing and then decreasing. Males showed a marked upward trend overall. For females, those born between 1990 and 1940 exhibited increasing risk, while those born after 1940 showed a decline. Sex-based differences also varied by birth cohort: among individuals born between 1900 and 1945, females had higher CVD mortality risk than males. In contrast, among later birth cohorts, males carried a higher risk (Figure 4C).

### 3.5 1990–2021 age-period-cohort (APC) model analysis of CVD DALYs attributable to high LDL-C in China

#### 3.5.1 Age effects

**Figure 5:**
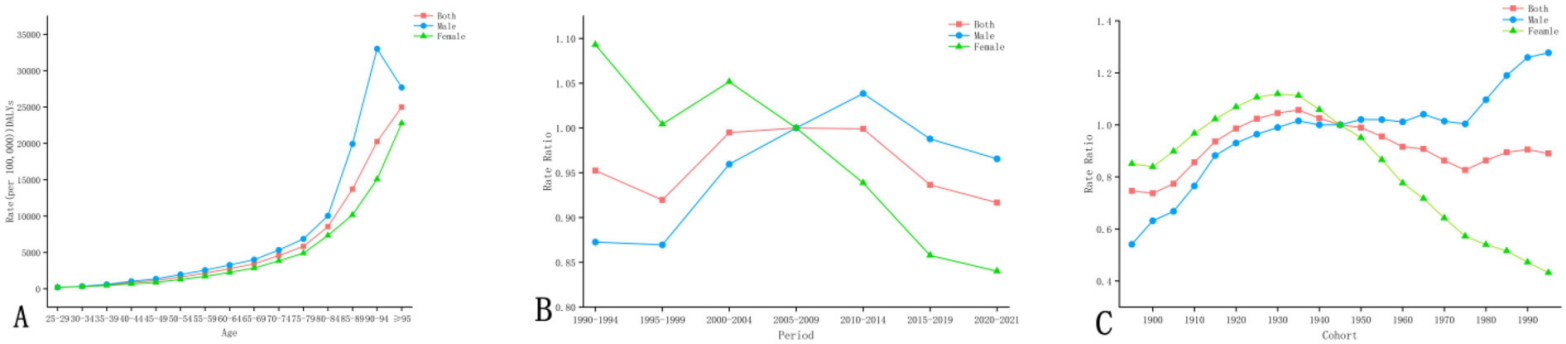
**(A)** Age, **(B)** period, and **(C)** cohort models of CVD DALYs attributable to high LDL-C in China, 1990–2021: overall, male, and female.

The rate of CVD DALYs attributable to high LDL-C increases with age, with a significant increase after 85 years of age. Among those aged 85–89, rates were substantially higher in males (e.g., 19,923.42 per 100,000) than in females (10,162.73) (Figure 5A).

#### 3.5.2 Period effects

The overall trend in DALY rates across different gender groups in China shows an initial increase followed by a decline. Males showed an initial increase followed by a decrease, while females experienced a pronounced decline (from 1.0931 to −0.8401). Sex disparities exhibit stage-specific characteristics: during 1990–2004, the CVD DALYs rate for the female population was relatively higher, while after 2009, the CVD DALYs rate for the male population was relatively higher (Figure 5B).

#### 3.5.3 Cohort effects

The CVD DALYs rate for different gender groups in China generally follows a pattern of first increasing, then decreasing, and then increasing again. Males showed a significant upward trend. Females born between 1990 and 1940 had increasing rates, while those born after 1940 experienced a decline. Further analysis revealed that there were stage-specific differences in DALY rates: among those born between 1900 and 1945, females had higher DALY rates than males. In subsequent birth cohorts, males exhibited higher rates (Figure 5C).

### 3.6 Predicted disease burden of CVD attributable to high LDL-C in China, 2022–2050, overall and by sex

**Figure 6:**
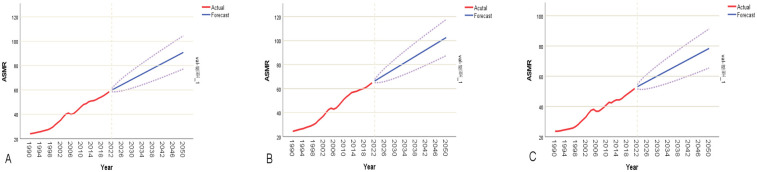
ARIMA model-based CVD mortality rate forecast for China (2022–2050). The red line represents the actual trend (1990–2021), and the blue line represents the future forecast, with a 95% confidence interval. **(A)** Shows the overall mortality rate forecast; **(B)** shows the male mortality rate forecast; **(C)** shows the female mortality rate forecast.

**Figure 7:**
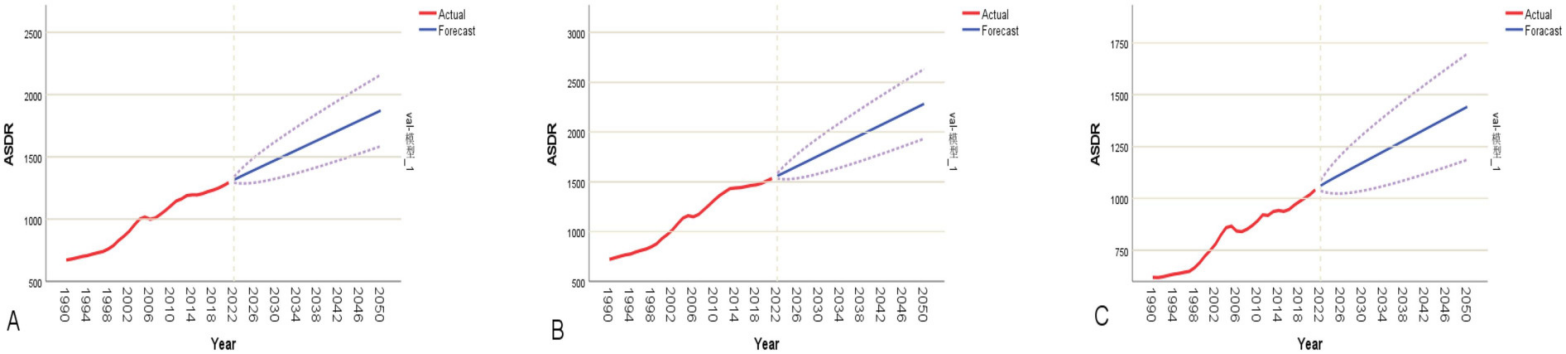
ARIMA model-based fitted predictions of CVD DALYs rates in China (2022–2050). The red line represents the actual trend (1990–2021), and the blue line represents the future forecast, with a 95% confidence interval. **(A)** Shows the fitted forecast for the overall DALYs rate; **(B)** shows the fitted forecast for the male DALYs rate; **(C)** shows the fitted forecast for the female DALYs rate.

Based on the ARIMA prediction model, the analysis reveals a relatively rapid growth pattern in CVD burden in China between 2022 and 2050. The model forecasts that by 2050, the overall ASMRs in China will rise to 90.82 per 100,000, representing a cumulative increase of approximately 55.14%. Sex-stratified analysis indicates that the ASMRs for males will increase from 64.85 to 102.60 per 100,000—an increase of 58.21%, which is slightly higher than the 51.07% increase observed in females (from 51.93 to 78.45 per 100,000). This suggests that the male population will continue to face persistent disease risk (Figure 6). The predicted overall ASDRs in China will rise from 1,293.82 to 1,873.18 per 100,000. The ASDRs for males is projected to increase from 1,534.25 to 2,283.25 per 100,000, while for females it will increase from 1,041.81 to 1,441.95 per 100,000 (Figure 7).

## 4 Discussion

### 4.1 Analysis of the burden of CVD in China caused by high LDL-C

This study comprehensively analyzed long-term trends in CVD mortality rates and DALYs attributable to high LDL-C in China from 1990 to 2021. The findings revealed a rapid upward trend in CVD mortality rates due to high LDL-C over the past 32 years, highlighting the urgent need for enhanced focus on the diagnosis and treatment of CVD in China. Additionally, significant gender and age disparities were noted, with males experiencing a higher burden compared to females. These results are consistent with a study on the CVD burden in ASEAN countries (15). The wide uncertainty intervals (UI) in the data reflect the multiple sources of uncertainty inherent in the estimation process of disease burden data obtained from GBD Study. These sources include data gaps, model assumptions, regional heterogeneity, and inconsistencies in the classification of causes of death, among others. This broad range of UIs exerts a certain impact on the reliability of research findings, which requires full consideration when formulating relevant health policies.

High LDL-C levels are a critical indicator for the prevention and treatment of CVD. Epidemiological studies consistently demonstrate a correlation between plasma LDL-C concentrations and the risk of atherosclerotic cardiovascular disease (ASCVD) events (16). A Mendelian randomization study revealed that reducing LDL-C levels decreases both the risk and mortality associated with CVD (17). In recent years, the prevalence of high LDL-C levels in China has risen due to various factors: economic development has increased the consumption of high-fat, high-sugar foods, Western fast food, and refined grains; a lack of physical activity and sedentary lifestyles contribute to obesity; and smoking and excessive alcohol consumption disrupt metabolic processes (18). The aging population, combined with decreased metabolic capacity and the prevalence of chronic diseases among the elderly, further exacerbates this issue. Additionally, air pollution, certain medications, and genetic factors contribute to the increasing burden of CVD (19).

### 4.2 Joinpoint regression analysis of the burden of CVD in China caused by high LDL-C

According to the analysis results of the joinpoint regression model, from 1990 to 2021, the ASMR and ASDR related to CVD caused by high LDL-C were higher in men than in women. Notably, the ASMR and ASDR in men exhibited an upward trend, while those in women showed a downward trend. Sex disparities in the disease burden of CVD caused by high LDL-C may be attributed to physiological factors. In premenopausal women, estrogen plays a protective role in the cardiovascular system by regulating nitric oxide release, mediating vasodilation, reducing atherosclerotic lesions, and improving endothelial function, thereby decreasing the incidence of cardiovascular disease (20, 21). Additionally, women and men exhibit significantly different lifestyles and health awareness: women generally have a stronger sense of health, whereas men are typically less health-conscious, pay less attention to their own well-being, and have insufficient awareness and concern regarding diseases. In traditional social concepts, men often assume the role of the family's pillar, facing greater work pressure and family burdens. This long-term high-pressure state may lead men to adopt unhealthy lifestyles more readily, such as staying up late, smoking, and drinking, all of which increase the risk of CVD (22). Men are also more likely to engage in binge drinking and prolonged alcohol consumption when dealing with stress (23). A prospective cohort study found that both long-term light drinking and moderate-to-heavy drinking are positively correlated with the risk of events related to cardiometabolic risk markers (24). Studies have confirmed that smoking increases the risk of cardiovascular disease by initiating oxidative stress, promoting inflammation, and abnormally activating the complement system in the immune system (25). Sex-based differences in pharmacological responses may represent a significant factor contributing to disparities in CVD burden. Research has revealed that statins, as cornerstone agents in lipid-lowering therapy, exhibit differences in therapeutic effects and safety profiles between sexes. Although women achieve LDL-C reduction of comparable magnitude to men following statin administration, they may be susceptible to higher risks of adverse drug reactions at equivalent dosages (26). Therefore, it is imperative to prioritize controlling CVD mortality and DALYs caused by high LDL-C in both men and women. Intensifying health education and promotion efforts, while fully considering the differences in behavioral patterns between men and women, is crucial for effective prevention and control measures and tailored interventions.

### 4.3 Age-period-cohort analysis of the burden of CVD in China caused by high LDL-C

Trends in mortality rates across various age groups, as revealed by age-specific mortality analyses, indicate minimal fluctuations in mortality rates for individuals under 65 years of age between 1990 and 2021. This suggests that CVD prevention efforts and early intervention measures targeting this age group have been effective. Data further show that mortality and DALYs rates attributable to high LDL-C for CVD among residents aged 25–95 years increase with age, with a significant rise observed after the age of 70. This increase may be attributed to physiological changes in the elderly, reduced vascular elasticity, declining physical condition, an increase in chronic underlying diseases, and lifestyle changes. This phenomenon may be attributed to the fact that individuals aged 70 and above often have multiple underlying conditions such as hypertension and diabetes, which synergize with elevated LDL-C to accelerate atherosclerosis and increase the risk of cardiovascular events. Older adults often take multiple medications, some of which may interfere with lipid metabolism or affect the efficacy of lipid-lowering drugs. Additionally, due to cognitive decline and mobility limitations, older adults exhibit lower disease screening rates and treatment adherence. Primary care settings also tend to adopt more conservative lipid-lowering treatment approaches for this population, leading to inadequate control of high LDL-C and further exacerbating the disease burden (27). The proportion of the population aged 65 and above rose from 5.6% in 1990 to 13.5% in 2020 and is projected to exceed 30% by 2050 (28). China's aging population presents increasingly severe public health challenges. As the aging process intensifies, the proportion of elderly individuals at elevated risk of LDL-C-related CVD will increase. Without enhancing lipid management and cardiovascular care for the elderly and implementing preventive measures, the future burden of high LDL-C-related CVD may further escalate. This underscores the urgency and importance of developing comprehensive health strategies for older adults and proactive lipid management programs.

The period effect refers to the impact of external factors on disease trends. This study's findings indicate that mortality and DALY rates rose variably between 1990 and 2009, potentially due to resource constraints during the early stages of public health system reforms post-2003 SARS epidemic, inadequate primary chronic disease management, low lipid screening and statin utilization rates, and dietary Westernization around 2010, characterized by increased red meat and processed food consumption, contributing to higher dyslipidemia rates. The decline from 2010 to 2021 can be attributed to the gradual expansion of medical insurance coverage (29). Additionally, advancements in medical technology, such as the use of statins and heightened public awareness of CVD, have positively influenced mortality and DALY numbers. Key time points between 1990 and 2021 coincide with significant milestones in China's medical insurance reform (30). In 1998, China introduced a basic medical insurance system for employees, integrating previously undiagnosed CVD patients into the healthcare system and boosting registration rates. However, the scarcity of lipid-lowering medications in primary care settings has caused treatment delays, intensifying the disease burden. Following the implementation of the Social Insurance Law of the People's Republic of China in 2010, the stability of the social insurance system improved. Consequently, statins, crucial CVD treatment drugs, were added to the essential drug list, lowering treatment costs and reducing the overall CVD burden (31).

This study encompassed 15 birth cohorts, each encountering unique risk factors. In China, the risk of CVD attributable to high LDL-C levels initially rose and subsequently declined across successive birth cohorts, notably after 1952. This trend is largely attributed to fluctuating social conditions. Women from birth cohorts prior to 1940 experienced a greater burden compared to men, likely due to malnutrition and compromised reproductive health during wartime. Conversely, men born after 1940 saw their risk continue to increase, potentially influenced by the pronounced impact of lifestyle changes associated with China's rapid economic development.

### 4.4 ARIMA prediction model analysis of the burden of CVD in China caused by high LDL-C

According to the ARIMA forecasting model, the disease burden of CVD attributable to high LDL-C levels in China from 2022 to 2050 is expected to continue its current trajectory. Notably, the ASMR and ASDR among males increased significantly more than those among females. The “Healthy China 2030” initiative aims to effectively manage major health risk factors by 2030. Consequently, greater emphasis should be placed on mitigating the CVD disease burden associated with elevated LDL-C levels, particularly through the implementation of targeted screening strategies for males. For men aged 35–64 years, LDL-C testing should become a mandatory component of routine physical examinations. Additionally, special initiatives for lipid management among the elderly should be conducted within communities, leveraging existing elderly care service facilities to establish ‘dietary intervention workshops.’ These workshops should develop low-cholesterol meal plans tailored to the dietary habits of older adults and promote appropriate physical activities, such as Tai Ji and Baduanjin.

This study has the following limitations: First, the research data relies on the 2021 GBD study, which itself is derived from model estimates. Furthermore, the GBD's LDL-C monitoring data originates from multiple countries, with significant variations in data coverage across different regions, potentially leading to biases in the estimated results. Additionally, over the 32-year period from 1990 to 2021, diagnostic criteria for cardiovascular diseases, coding standards, and LDL-C testing methods may have evolved. These technical changes were not incorporated into the analysis, potentially affecting the accuracy of disease burden trends. Second, due to data availability constraints, this study could not conduct comparative analyses of disease burden trends across Chinese provinces or between urban and rural areas, limiting the ability to reveal regional-level variations. Finally, the disease burden projections for 2022–2050 employed an ARIMA model, which was constructed based on historical trends and assumed no changes in current public health policies or interventions. This assumption disregarded potential future policy adjustments or intensified interventions, potentially underestimating the mitigating effects of proactive interventions on disease burden. This study did not conduct sensitivity analyses or scenario simulations (e.g., interventions such as “increased LDL-C screening rates”), limiting the robustness of the projections and their policy reference value.

## 5 Conclusion

From 1990 to 2021, the disease burden of CVD attributable to high LDL-C in China exhibited an upward trend, marked by notable demographic disparities. While the disease burden among males has consistently increased, it has shown a declining trend among females, with the elderly population experiencing the most substantial rise in burden. As the rate of population aging accelerates, this disease burden is anticipated to worsen further. Projections suggest that by 2050, the disease burden of CVD due to high LDL-C levels will continue to escalate, with men remaining a high-risk group necessitating focused attention. A comprehensive intervention system must be established, emphasizing risk prevention and control for men, enhancing lipid management for the elderly, optimizing resource allocation at the grassroots level to improve early intervention capabilities, and developing a national cholesterol control strategy to standardize drug therapy. Additionally, public health initiatives should be promoted to expand LDL-C screening and improve access to lipid-lowering medications to address cardiovascular health issues.

## Data Availability

The datasets presented in this study can be found in online repositories. The names of the repository/repositories and accession number(s) can be found in the article/Supplementary Material.
